# From rubber hands to neuroprosthetics: Neural correlates of embodiment

**DOI:** 10.1016/j.neubiorev.2023.105351

**Published:** 2023-10

**Authors:** Fabio Castro, Bigna Lenggenhager, Daniel Zeller, Giovanni Pellegrino, Marco D’Alonzo, Giovanni Di Pino

**Affiliations:** a Neurophysiology and Neuroengineering of Human-Technology Interaction Research Unit, Campus Bio-Medico University, via Alvaro del Portillo 5, 00128 Rome, Italy; b Institute of Sport, School of Life and Medical Sciences, University of Hertfordshire, Hatfield, United Kingdom; c Department of Psychology, Cognitive Psychology, University of Konstanz, Universitätsstraße 10, 78464 Konstanz, Germany; d Department of Psychology, University of Zurich, Binzmuehlestrasse 14, 8050 Zurich, Switzerland; e Department of Neurology, University Hospital Würzburg, Josef-Schneider-Str. 11, 97080 Würzburg, Germany; f Epilepsy program, Schulich School of Medicine and Dentistry, Western University, London, Ontario, Canada

**Keywords:** Rubber hand illusion, Embodiment, Prosthesis, Amputation, Intersensory conflict, Bodily self, Multisensory integration

## Abstract

Our interaction with the world rests on the knowledge that we are a body in space and time, which can interact with the environment. This awareness is usually referred to as sense of embodiment. For the good part of the past 30 years, the rubber hand illusion (RHI) has been a prime tool to study embodiment in healthy and people with a variety of clinical conditions. In this paper, we provide a critical overview of this research with a focus on the RHI paradigm as a tool to study prothesis embodiment in individuals with amputation. The RHI relies on well-documented multisensory integration mechanisms based on sensory precision, where parietal areas are involved in resolving the visuo-tactile conflict, and premotor areas in updating the conscious bodily representation. This mechanism may be transferable to prosthesis ownership in amputees. We discuss how these results might transfer to technological development of sensorised prostheses, which in turn might progress the acceptability by users.

## Highlights

• The rubber hand Illusion (RHI) is used to investigate embodiment.
• Visuo-tactile conflict induced by the RHI is resolved by multisensory areas.
• These areas attenuate somatosensory information, in favour of visual one.
• Embodiment in amputees relies on similar neurophysiological mechanisms.
• RHI findings can be translated to design sensorised prostheses easier to embody.

## 1 Introduction

**Fig. 1:**
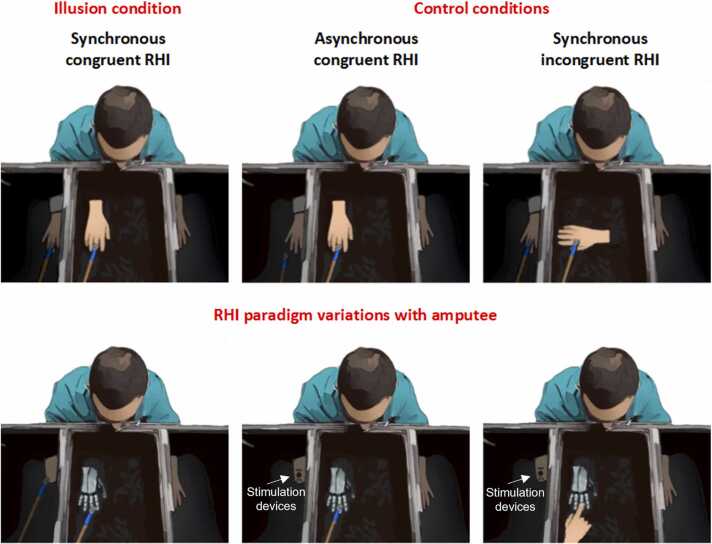
Schematic representation of the RHI protocol in healthy and amputees. In the synchronous condition, participants watch the experimenter bushing the rubber hand and the real hand simultaneously. In the asynchronous condition, the experimenter brushes the real and the dummy hand with a delay, such that the participant feels the brushing on the real hand when the rubber hand is not brushed. This creates a visuo-tactile conflict. In the incongruent condition the brushing is done synchronously, but with the rubber hand in a an anatomically implausible position, usually rotated 90 or 180 degrees from the congruent condition. In amputees, stimulation is done at the stump, where finger sensation is referred, by using a paintbrush or invasive or non- invasive stimulation devices. The visible stimulation on the prosthetic or robotic hand can be brushstrokes or touch.

How does it feel to be or own a body? Typically, when looking at our hands, we immediately and effortlessly perceive them as part of our body (*embodiment*), a feeling that includes the sense of ownership (“This hand is mine”), the sense of agency (“It was me who moved that hand”) and the self-location (“My hand is there”; Kalckert and Ehrsson, 2012; Longo et al., 2008). Although not all researchers agree on their appropriateness to study multisensory mechanisms underlying body perception (Dieguez, 2018, Lush, 2020), hitherto embodiment has been largely investigated through bodily illusions (Botvinick, 2004). An interesting feature of illusions (including bodily ones) is the creation of a conflict between expectancy-based priors and incoming sensory information (Adams et al., 2013, Gregory, 1980, Shipp et al., 2013). One of the best-known bodily illusions is the rubber hand illusion (RHI; Botvinick, M. and Cohen, J. D, 1998). In a typical RHI paradigm, the experimenter brushes simultaneously a visible dummy hand, and the participant’s hidden hand (Botvinick, M., and Cohen, J. D, 1998; Fig. 1). When the stroking of the rubber and the real hand is performed congruently in time (synchrony) and in space (matching hand-brushing orientation and location), the dummy hand is embodied, meaning that it is perceived as part of one’s body (Botvinick, M., and Cohen, J. D, 1998). Over the past twenty years variations of the paradigm have been proposed in the attempt to investigate fine-graded aspects of RHI and thus the sense of embodiment. These variations in the RHI can be broadly classified in four groups: i) presentation methods, i.e., the methods used to present the rubber hand (e.g., virtual vs rubber hand); ii) assessment methods, i.e., the subjective and objective indices used to measure embodiment; iii) illusion induction method, i.e., the stimulation procedure to induce the visuotactile conflict (e.g., brush stroking vs vibration); and iv) control conditions, i.e., the conditions to which the illusion paradigm is compared (for an in-depth discussion on the topic, see Riemer et al., 2019).

Bodily illusions, especially the RHI, are thought to arise from complex integration processes between multisensory information and expectations about sensory information (Tsakiris, 2017). At a mechanistic level, this interaction has been suggested to follow Bayesian inference. Within this umbrella concept, different theories explain how multisensory integration may be implemented by the brain (Körding et al., 2007, Limanowski, 2021, Sato et al., 2007). In the RHI, the sensory conflict created by the experimental setup highlights the importance of multisensory integration in body perception and action, whose underlying principle is thought to be sensory weighting based on epistemic value (Parr and Friston, 2017). Such integration may be conceptualized as the ability of the brain to resolve the conflict among sensory information, based on precision of the sensory source (Limanowski, 2021). This mechanism is thought to be grounded on hierarchical computational messages passing between lower-level areas, which process fast-changing physical aspects of perception and action, and higher-level multisensory areas, which integrate and process slower-changing and increasingly abstract representations of the body and its interaction with the environment (Limanowski, 2014). In another theoretical framework, Bayesian Causal Inference, which recently has been extended to the rubber hand illusion, multisensory integration is based on a generative model based on spatial proximity, simultaneity, temporal correlation, sensory uncertainty and prior perceptual experience (Chancel et al., 2022, Chancel et al., 2022, Kilteni et al., 2015, Samad et al., 2015).

In addition to reveal fundamental mechanisms of embodiment in healthy participants, the RHI procedure has been useful in patient populations (Fiorio et al., 2011, Lenggenhager et al., 2012, Lenggenhager et al., 2013, Zeller et al., 2011) for understanding disorders of the sense of embodiment, as well as for the development of potential therapeutical applications (Lenggenhager et al., 2014). An interesting population in this regard are amputees, where the successful embodiment of an artificial limb, i.e., prothesis, has shown to be beneficial inducing also reduction in aberrant plasticity (Di Pino et al., 2009). The application of the RHI procedure has been used to investigate to what extend amputees are able to embody such foreign and artificial body parts. A successful RHI in people with amputation can be not taken for granted, as studies suggest that amputations result in quite dramatic neural plasticity leading to a redistribution of neural stations and connections to remaining body parts (Di Pino et al., 2009, Muret and Makin, 2021), which might alter many behavioural, physiological and phenomenal processes (Makin et al., 2017). Importantly, perceiving the prosthesis as foreign is one of the reported causes of its rejection (Murray, 2004), which is a common issue among amputees. In the following sections we first review neural and physiological correlates of the RHI in healthy individuals, and then we attempt transposing these insights to the field of neuroprosthetics. The overall aim of this paper is to highlight neuronal and computational underpinnings of embodiment, and how this may fundamentally contribute to the development of restorative technologies.

## 2 Neuroimaging studies on the rubber hand illusion

Most studies investigating neural correlates of the RHI used fMRI. A consistent and convergent finding thereof is that embodiment during the RHI is correlated with activity changes in a network including parietal – posterior parietal cortex (PPC) and inferior parietal sulcus (IPS) – and premotor areas, notably the ventral premotor cortex (PMv) (Bekrater-Bodmann et al., 2014, Brozzoli et al., 2012, Ehrsson et al., 2004, Petkova et al., 2011). These areas are part of the dorsal stream (Goodale, 2011, Milner and Goodale, 2008), which is crucially involved in sensorimotor transformation for action (Cisek and Kalaska, 2010). Indeed, the activity of the frontoparietal network has been linked to action preparation (Davare et al., 2011, Jeannerod et al., 1995, Pellegrino et al., 2018, Tombini et al., 2009), action understanding (Rizzolatti and Craighero, 2004), and internal simulation of actions (Hardwick et al., 2018, Jeannerod, 2001). Together, parietal and premotor areas integrate visual and somatosensory information thanks to multimodal neurons encoding and constantly updating participants’ corporeal space (Braun et al., 2018). Parietal cortex is a major hub for multisensory integration (Fogassi et al., 2005), and contributes to map position and orientation of limbs in space within a body-centred reference frame (Grivaz et al., 2017). Moreover, the PMv contains neurons with visual and tactile receptive fields that respond whenever a specific body part is touched or a stimulus is seen approaching this specific body region (Botvinick, 2004, Gentile et al., 2013). Premotor activity during RHI suggests a shift in neural processing of participants’ hand, becoming aligned with the dummy hand (Botvinick, 2004). In line with this tenet, a stronger perception of the RHI has been linked to increased activity in PMv ( (Ehrsson et al., 2004, Gentile et al., 2013, Petkova et al., 2011). PMv activity is sensitive to time congruency: a delay between seen and felt brushstrokes ranging between 0 and 300 ms is optimal to induce the illusion and did not produce different brain activity, while 0 ms compared to 600 ms inter-brushing interval resulted in increased activity in contralateral (right) PMv and ipsilateral (left) inferior parietal cortex (Bekrater-Bodmann et al., 2014).

**Fig. 2:**
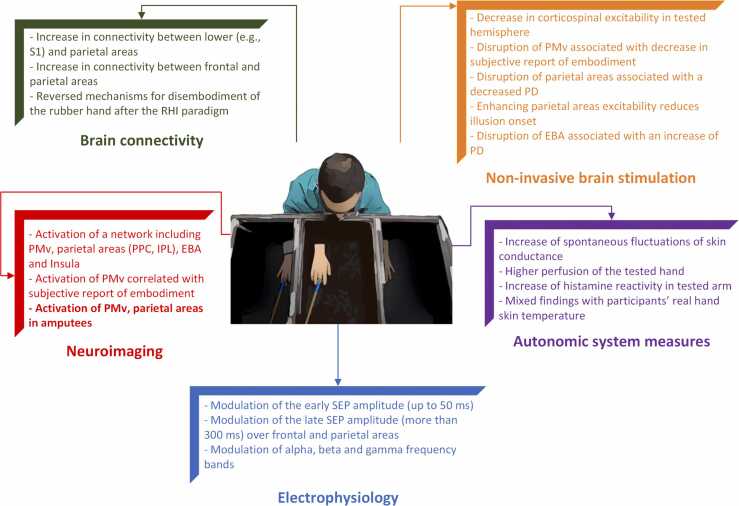
Schematic summary of the main results on the neural correlates of the RHI, divided by techniques. The results obtained in population with amputation in bold.

While premotor and parietal areas are consistently involved in the RHI, additional brain areas are active only in specific experimental conditions. In a study, threatening the rubber hand induced increased activity in the insula (Ehrsson et al., 2007), which may be related to interoceptive and emotional processing linked to bodily perception (see section ‘Autonomic Correlates to the RHI’). Other studies also found activity in the lateral occipital cortex (LOC), specifically extrastriate body area (EBA) is linked to altered embodiment during the RHI (Gentile et al., 2013, Limanowski and Blankenburg, 2015). This area, part of the visual system, has preferential tuning towards visual processing of body parts (Downing et al., 2001, Pitcher et al., 2009). EBA activity during RHI has been proposed to reflect interindividual differences in the intensity of the illusion (Limanowski et al., 2014). These main findings are summarized in Fig. 2 (Neuroimaging).

## 3 Electrophysiological correlates of the rubber hand illusion

To assess the temporal dynamics of brain activity induced by the RHI, several studies have used electroencephalography (EEG). Studies using somatosensory evoked potentials (SEP) triggered by brushstrokes suggest that the RHI is associated with modulation of both early and late potentials. Decreased SEP amplitude with a frontal negative peak over left frontal area was found around 50 ms in the illusory, i.e., congruent in position condition, while a larger parietal positive peak emerged around 50 ms in the incongruent condition (Zeller et al., 2015). This may point to an early attenuation of proprio- and exteroceptive inputs within the primary somatosensory cortex (S1) and sources in the anterior intraparietal sulcus in the illusory condition, even before processing at higher hierarchical levels occurred. Another study found modulated SEP amplitudes over the central region contralateral to the stimulated hand (roughly corresponding to sensorimotor areas) at later latencies. Synchronous stimulation resulted in a significant increase in SEP amplitude at 150 ms, and a significant decrease 460 ms after the stimulation, compared to pre-illusion condition. In addition, ipsilateral SEP amplitude was increased between 80 and 100 ms (Peled et al., 2003), probably reflecting illusion-related processes within associative regions. Taken together, electrophysiological evidence supports early attenuation of somatosensory input prior to signal modulation within multimodal, associative brain regions associated with the illusory percept of owning an artificial hand.

Collecting SEPs during RHI induction poses several methodological challenges: As opposed to the electric pulses typically employed in SEP, brushstroking is not a short-lasting discrete event. Recently, by alternating brushstrokes to induce the RHI and median nerve electrical stimulation to evoke SEP, significantly lower N1-P1 (peaking at approximately 20–25 ms) component was found in the synchronous condition (Sakamoto and Ifuku, 2021). Note that N1-P1 is the earliest reliable SEP component, reflecting somatosensory processing in the contralateral sensorimotor regions (Hashimoto et al., 1990). In an additional experiment to investigate illusion-specific effects, the same authors instructed participants to push a button to indicate illusion onset. In line with previous results (Zeller et al., 2015), the amplitude of the N1-P1 component was significantly smaller both before and after the onset of the illusion, compared to rest.

Applying a more sophisticated set of experimental conditions to control for potential confounds, Rao and Kayser observed an illusion-related attenuation of event-related potentials in frontocentral areas and a decrease of frontal alpha and beta power around 330 ms, thus referring to late rather than early higher-order sensory processes (Rao and Kayser, 2017, Niso et al., 2021). In contrast, a recent fMRI study described increased activity in S1 during illusory body ownership which was even enhanced when the sensation of agency was added (Abdulkarim et al., 2023). A further SEP study using multimodal stimulation (visual or vibrotactile or combined stimulations) to induce the RHI, linked the N140 (i.e., negative potential highlighted around 140 ms post stimulus), which especially in its higher frequency components maps to the secondary somatosensory cortex (S2), to the multisensory integration attentional process (Kanayama et al., 2007).

Taken together, electrophysiological evidence paints an ambiguous picture of signal modulation within multimodal, associative brain regions associated with the illusory percept of owning an artificial hand. The particular role of a modulation of early and late components by the RHI remains open. So far, methodological aspects regarding stimulus application and choice of control conditions may have limited comparability and generalizability of findings. Thus, further studies with best possible control for these aspects are needed.

Studies have also investigated RHI-induced modulation of brain activity across frequency bands, which are thought to reflect different computational and neurophysiological mechanisms (Bastos et al., 2012, Limanowski et al., 2020, Palmer et al., 2019). Modulation in alpha, beta and gamma frequency bands has been reported. Visuotactile processing during congruent and incongruent multimodal stimulation was associated with increased activity in parietal areas in the gamma (25–35 Hz) frequency band after 200–350 ms (Kanayama et al., 2007, Kanayama et al., 2009), and 500–700 ms after multimodal stimulation (Kanayama et al., 2007). Increased activity in gamma frequency band has long been associated with multimodal processing (Eimer et al., 2004, Forster and Eimer, 2004, Forster and Eimer, 2005), so it is possible that these modulations over parietal areas reflect a multisensory integration process, in line with fMRI studies (see previous sections). Another study reported modulation in alpha (8–12 Hz) frequency band over central and parietal electrodes, and beta (13−25) frequency band over fronto-parietal electrodes in the window between 100 ms pre-stimulus and 300 ms post stimulus (Rao and Kayser, 2017), suggesting a role of these areas in mediating illusory embodiment.

A single experiment with intracranial recordings performed during the pre-surgical evaluation of five epileptic patients reported a differential role of premotor and parietal cortices in the RHI (Guterstam et al., 2019). Synchronous stroking was associated with increased activity in high gamma frequency bands in IPS and premotor cortex (PMC), but not in S1, both during stroking and in between stroking (c.f. Sakamoto and Ifuku, 2021). Interestingly, when inter-areas connectivity distance was accounted for, by shifting offline forward by 200 ms the ECoG trace recorded from the somatosensory cortex, IPS, but not PMC activity was significantly modulated by tactile information. This was also corroborated by an increased connectivity between S1 and IPS. 200 ms has been reported to be the time for tactile information to reach multimodal areas (Duhamel et al., 1998), and this further corroborates connectivity studies (see below for more details) suggesting that embodiment of the rubber hand is at least initially encoded by messages passing between lower-order sensory areas and the parietal cortex, with premotor areas involved at later stages. Together, these studies provide evidence that, during the RHI, brain activity may be modulated in two phases: an early activity over parietal and somatosensory cortex, and a later activity in more anterior areas, including the central and frontal regions. However, the heterogeneity of the experimental setups and analyses makes direct comparison between the studies difficult. As an interesting finding with direct significance for the embodiment of prostheses (see below), studies also suggest that embodiment of the dummy hand may emerge as sustained multisensory integration which modulates activity between areas involved in body representation (Guterstam et al., 2019, Sakamoto and Ifuku, 2021, Zeller et al., 2015, Zeller et al., 2016). These main findings are summarized in Fig. 2 (Electrophysiology).

## 4 Network-level changes induced by the rubber hand illusion

The neural correlates of the RHI are certainly ascribed to the functional specialization and segregation of sensorimotor and cognitive areas, but they are represented as well by the functional integration among these brain areas, which is investigated with connectivity analyses (c.f. Herbet and Duffau, 2020). Limanowski and Blankenburg (2015) were among the firsts to study bold-related network-level modulations induced by RHI. Consistent with studies discussed so far, synchronous vs asynchronous stimulation resulted in enhanced functional connectivity between left PMv, IPS, S2 and EBA. Dynamic causal modelling (DCM) – a technique used to model effective connectivity (Friston et al., 2017) – indicated that during congruent brush stroking there was an increased connectivity between S2 and the lateral occipital cortex towards IPS. This finding complies with the idea that during illusory self-attribution, unpredicted ambiguous sensory input may generate prediction errors in visual and somatosensory areas, which may be conveyed to parietal integrative areas (Limanowski and Blankenburg, 2015). Another fMRI study explored the mechanisms of “recovery” from the RHI, i.e., the neural correlates of sudden loss of the illusion (Lee and Chae, 2016). Connectivity changes were investigated when acupuncture was provided either as a tactile stimulus on the real, or as a visual stimulus on the rubber hand, each of which is known to re-instantiate limb ownership immediately after RHI-induced “disownership”. For disruption of the RHI by pricking the real hand, DCM analysis revealed decreased connectivity between IPS and S2 as well as IPS and EBA. On the contrary, connectivity between IPS and PMv increased. Visually stimulating the rubber hand was associated with an increase of effective connectivity between IPS and LOC, along with a decrease between IPS and S2. Taken together, these studies shed light on how the brain solves the intermodal conflict associated with the RHI. Other studies explored RHI-related connectivity using EEG. In a DCM connectivity study, intrinsic connectivity in S1 was found to be lower during congruent stimulation, i.e., during illusory perception, along with increased connectivity between occipital and premotor areas (Zeller et al., 2016). This was interpreted as the result of an attenuation of somatosensory processing. It is worth noting that these results are not completely in line with those reported by Limanowski and Blankenburg (2015), which are based on a different modelling approach (Friston et al., 2017, Kiebel et al., 2009).

Recently, another study found decreased connectivity in EEG high-alpha and beta frequency bands between medial and parietal areas about 200 ms after congruent stimulation (Kanayama et al., 2017). This was negatively correlated with the referral of touch (“*it seems as if I was feeling the touch of the paintbrush in the location where I saw the rubber hand touched*”) and the ownership (“*I felt as if the rubber hand was my hand*”) items of the RHI questionnaire (Table 1). Furthermore, the authors also found increased connectivity from left parietal towards right somatosensory areas between 550 ms and 750 ms after the brushstroke, which positively correlated with the proprioceptive drift (PD: i.e., the shift of the perceived hand location towards the rubber hand due to the RHI paradigm administration). This suggests that the proprioceptive drift and the subjective experience of the RHI, albeit correlated between them (Botvinick and Cohen, 1998, Tosi and Romano, 2022), partially rely on different processing, as already shown by several studies based on behavioural measures (e.g., Abdulkarim and Ehrsson, 2016, Erro et al., 2018, Rohde et al., 2011).

**Table 1:** List of items of the questionnaire (between parentheses the terms to substitute when the questionnaire is administrated to an amputee). Three items (i.e. illusion items) refer to the extent of sensory transfer into the rubber hand and its self-attribution during the trial. The other six items (i.e. control items) serve as controls for compliance, suggestibility, and “placebo effect”. In addition, the vividness and prevalence statements are reported.

Questionnaire	Item	Rating
Item 1	It seemed as if I were feeling the tactile stimulation at the location where I saw the visible hand touched	-3 – + 3
Item 2	It seemed as though the stimulation I felt was caused by the touch on the visible hand	
Item 3	I felt as if the visible hand was mine	
Item 4	I felt as if the position of my real hand (stump) was drifting towards the visible hand	
Item 5	It seemed as if I had more than two (one) hand or arm	
Item 6	It seemed as if the tactile stimulation I was feeling came from somewhere between my own hand (stump) and the visible one	
Item 7	I felt as if my real hand (stump) were turning ‘rubbery’	
Item 8	It appeared as if the position of the visible hand was drifting towards my real hand (stump)	
Item 9	The visible hand began to resemble my own hand (stump), in terms of shape, skin tone, freckles or some other visual features	
Vividness	How realistic and life-like was the illusion that the visible hand was yours when it was experienced?	0 – 10
Prevalence	How long with respect to the length of section was the perception of this illusion?	0 – 100%

Taken together, fMRI and EEG studies confirm that the process of embodiment involves the modulation of networks, such as the connectivity between early sensory-related areas, multisensory parietal areas, and premotor cortices to resolve the multisensory conflict. Similar, but opposite, mechanisms also seem to be involved to re-establish the ownership of the real hand (Fig. 2 Brain connectivity).

## 5 Non-invasive brain stimulation

Non-invasive brain stimulation techniques allow to interfere with ongoing neural activity, and thus to reveal a causal role of the stimulated area in perception or behaviour. Transcranial magnetic stimulation (TMS) and transcranial direct current stimulation (tDCS) have been used extensively to study the neural mechanisms underlying the RHI. Applying a single TMS pulse to primary motor cortex (M1), the resulting motor-evoked potentials (MEPs) recorded from a target muscle can be used as a readout of corticospinal excitability at rest (Bestmann et al., 2015, Rossini, 2015), during perceptual (Castro et al., 2021, Pellegrino et al., 2022) and motor tasks (Castro et al., 2021, Pascual-Leone et al., 1995). The involvement of corticospinal neurons is thought to be mostly indirect, via intracortical and cortico-cortical connections, depending on the coil orientation and stimulation intensity (Di Lazzaro et al., 2012).

Studies report contrasting results bodily illusion, including the RHI (Dilena et al., 2019). During the RHI, MEP amplitude have been reported to be significantly lower, compared to asynchronous stroking or baseline (Schütz-Bosbach et al., 2006, Schütz-Bosbach et al., 2009), lateralised to the touch-stimulated hand (della Gatta et al., 2016). Furthermore, della Gatta and colleagues reported a temporal interrelationship between synchronous stroking and MEP amplitudes, such that the longer the synchronous stroking period, the lower the MEP amplitude (della Gatta et al., 2016). A suppression of corticospinal excitability has also been reported during observation of self-attributed actions after the RHI. When people observed index finger adduction/abduction during the synchronous RHI condition, MEP amplitude in the first dorsal interosseous muscle (i.e., the target muscle) was significantly lower compared to observation of the same action during asynchronous stroking (Schütz-Bosbach et al., 2006). Interestingly, modulation of corticospinal excitability showed topological specificity, as no MEP changes were found in the Abductor digiti minimi muscle, which was not involved in the observed action. In addition, the GABAergic suppression of voluntary EMG activity after a contralateral TMS pulse was significantly increased after synchronous stroking (Schütz-Bosbach et al. (2009), thus suggesting a neurophysiological mechanism that potentially correlates with the emergence of the illusion. It should be noted, however, that other studies question the validity and reliability of corticospinal excitability as a measure of embodiment. Indeed, no changes in MEP amplitude have been found for the motor version of the RHI where participants moved the hand and saw the rubber hand reproducing the same movement. Furthermore, recent evidence failed to reproduce the results of della Gatta and colleagues (Reader et al., 2021). Together, these results do not allow robust conclusion on the effect of the RHI on corticospinal excitability, and further studies are needed. Noteworthy is the fact that most studies in this area do not use the same methodology, which may have influenced results. It is possible that the effect of the RHI on the motor cortex is rather small, and may be masked by small methodological differences. Further studies are needed to evaluate the robustness and reliability of the M1 activation during the RHI.

TMS studies discussed so far stimulated M1, which is connected to a wide network of cognitive and motor areas, including S1 and premotor cortex, both ventral and dorsal portion (Davare et al., 2011). Each of these regions has been suggested to play a role in the RHI (see earlier section for more on this). To assess the influence of those areas on corticospinal excitability, studies have used paired-pulse stimulation paradigms, delivered with single or double coil protocols. These paradigms investigate cortico-cortical and peripheral contribution to corticospinal excitability (Neige et al., 2021), and can provide insight into the neurotransmission pathway involved. Isayama et al. (2019) explored the effect of the afferent stimulus on M1 excitability with the short- and long-latency afferent inhibition paradigms (SAI and LAI, respectively), while connectivity between superior parietal lobule (SPL) and M1 was assessed using a double-coil stimulation paradigm. Both SAI and LAI were significantly reduced immediately after synchronous stimulation (RHI condition) as compared to asynchronous stimulation. Connectivity between SPL and M1 was not modulated by the RHI (i.e., no modulation of conditioned corticospinal excitability), but stronger illusion during the RHI correlated with conditioned MEP amplitude in synchronous, but not in asynchronous condition. Reduced SAI, which assesses the connectivity between somatosensory and motor areas (Turco et al., 2018) is in line with neuroimaging and EEG studies reporting an attenuation of sensorimotor areas, to resolve the visuotactile conflict arising from the stimulation, thus inducing the illusion (Limanowski and Blankenburg, 2015, Zeller et al., 2015). These main findings are summarized in Fig. 2 (Non-invasive brain stimulation).

### 5.1 Neuromodulation of RHI-related network

Non-invasive brain stimulation can be used to enhance or downregulate the excitability and to generate a transient ‘virtual’ lesion in target areas (Cirillo et al., 2016). Virtual lesions depend on the interference between the artificial stimulation and the spontaneous activity and they are employed to provide evidence of the involvement of the disrupted area in some aspect of behaviour (Pascual-Leone, 2000). Furthermore, non-invasive brain stimulation can also be used to correct the brain activity altered by a pathological condition (Di Pino et al., 2014). To the best of our knowledge, only one TMS study aimed to enhance excitability, while most studies used off-line low frequency TMS pulse trains (1 Hz) to downregulate a target area. Targeting IPL and PMv seems to differentially influence the measures of the illusion. 1 Hz rTMS over IPL administered to healthy participants shortly before synchronous stroking attenuated proprioceptive drift, but did not modulate subjective sense of ownership as measured by questionnaires (Kammers et al., 2009). Somewhat different results were obtained more recently applying the same stimulation (1 Hz rTMS) over PMv, compared to stimulation of the vertex (control site). Stimulation over PMv resulted in lower ratings for illusion-related items of the RHI questionnaire only in the synchronous condition. No effects of rTMS or stimulation site on proprioceptive drift was found (Peviani et al., 2018). This differential effect of the same rTMS protocol when delivered over PMv or IPL is, once again, suggestive of different neural mechanisms involved in proprioceptive drift and the phenomenal sense of ownership assessed by the questionnaire. Further evidence in this direction comes from tDCS studies. During tDCS a low-amplitude constant electric current flows between electrodes placed on the scalp (Nitsche and Paulus, 2000) and produces polarity specific effects, with inhibition of the cortex under the cathode and excitation of the region under the anode. Anodal tDCS over PPC, but not over PMv, decreases the onset time of the RHI (Lira et al., 2018), implying that upregulation of PPC makes RHI build-up faster. However, another study where the activity of PMv or IPL was enhanced via intermittent theta burst (iTBS) stimulation, a popular excitability enhancing TMS protocol (Huang et al., 2017), did not result in modulation of the RHI questionnaire and proprioceptive drift (Mioli et al., 2018). Multiple reasons may explain these differences, for instance the different mechanisms of action of tDCS and iTBS.

Studies targeted other cortical areas involved in the RHI network. 1 Hz rTMS over EBA induced an increased proprioceptive drift, with no effect on illusion onset or phenomenal ownership rating (Wold et al., 2014). Dissociation of behavioural measures was reported in another study (Convento et al., 2018); when the right temporoparietal junction (TPJ) was targeted by anodal tDCS, proprioceptive drift increased, while targeting PMC decreased this measure. In line with studies highlighting the role of the somatosensory cortex in the resolution of a visuo-proprioceptive conflict, cathodal tDCS of S1 was associated with significantly higher indices of embodiment of the rubber hand compared to anodal tDCS (Hornburger et al., 2019). There is also evidence that M1 may be involved in the RHI. Compared to sham stimulation, 1 Hz rTMS over right or left M1 increased proprioceptive drift as well as indices reflecting embodiment of the dummy hand and disembodiment of the real hand (Fossataro et al., 2018). Interestingly, MEPs were influenced by stimulation type of rTMS (sham vs. real), but not by the RHI condition (i.e., synchronous and asynchronous stimulation), suggesting a more general effect of rTMS on intrinsic corticospinal excitability.

Taken together, results from non-invasive brain stimulation studies expand those from studies using other techniques. Most notably, the same 1 Hz rTMS protocol over premotor or parietal areas – two areas that are consistently reported in fMRI studies – results in a different effect on RHI-related indices of altered body ownership, and this is in line not only with psychometric studies (Longo et al., 2008), but also with invasive electrophysiological data (Guterstam et al., 2019) (Fig. 2 Non-invasive brain stimulation).

## 6 Autonomic correlates of the RHI

Embodiment of non-body objects has also been associated with changes in interoceptive processing (Seth, 2013). This is not surprising, considering that pathways and cortical centres of interoceptive and exteroceptive processing often overlap (Follett and Dirks, 1994, Levinthal and Strick, 2012, Rebollo et al., 2018). Interoceptive afferents target the insula, via the thalamus, and have been shown to modulate internal representation of the body and the environment (Fermin et al., 2022). The RHI has been used to investigate the link between internal representation of the body and various autonomic indices. Synchronous stroking has been reported to decrease participants’ real hand skin temperature (Llobera et al., 2013, Moseley et al., 2008, Tieri et al., 2018, Tsakiris et al., 2011), even though the consistency of such finding is currently not confirmed by other studies (de Haan et al., 2017, Rohde et al., 2013) (Lang et al., 2021; Roel Lesur, Weijs et al., 2020). Furthermore, an increase of histamine reactivity was also observed by comparing wheal dimension after histamine injection on the tested and contralateral arm (Barnsley et al., 2011). Together, both selective cooling of the tested hand and the increase in histamine reactivity measured after the induction of the RHI may be related to a sense of disownership and as a sign of ‘rejection’ of the real hand in favour of the dummy limb. Similarly to downregulation of somatosensory and motor processes described previously, this suggests downregulation of homeostatic processes during the embodiment of the fake hand (Burin et al., 2017, della Gatta et al., 2016, Fossataro et al., 2018, Zeller et al., 2015, Zeller et al., 2016).

Skin conductance response to a threat has been used as an implicit objective tool to assess how much the fake hand in the RHI is embodied because, similarly to a startle reflex, physical threats to an owned body part evoke a stronger autonomic reflex: the engagement of emotional defense reactions (Armel and Ramachandran, 2003, Graziano and Cooke, 2006). Several studies found significant higher magnitude in threat-evoked SCR when embodying a fake body with respect to a control condition (Fan et al., 2021, Guterstam et al., 2020, Tacikowski et al., 2020, Tacikowski et al., 2020). Such measure has been also used to probe embodiment of prosthetic limbs (Ehrsson et al., 2008b). The magnitude of threat-evoked SCR has also been compared directly with brain responses (PMv activity correlates with threat-evoked SCR; Gentile et al., 2013). A correlation between the feeling of ownership of the artificial hand and the threat-evoked neuronal responses in the areas reflecting anxiety was also identified (i.e., anterior insula and the medial anterior cingulate cortex). Interestingly, threating the rubber hand can induce a similar level of activity in these brain areas as when the person's real hand is threatened (Ehrsson et al., 2007). However, it is not always found a correlation between the threat-evoked SCR values and subjective ratings (Mattsson et al., 2022, Roel Lesur et al., 2020, Weijs et al., 2021)*.* Interestingly, synchronous RHI brush-stroking enhances also spontaneous fluctuations of skin conductance, which correlates with illusory body ownership as measured by the questionnaire (D’Alonzo et al., 2020). Additionally, the perfusion of the tested hand was higher during synchronous stroking than during a control condition, as revealed by the brachial artery blood flow recorded during RHI (Di Pino et al., 2022). The blood flow value correlated with the degree of embodiment measured by self-assessment questionnaire. Moreover, artificially-induced peripheral ischemia modulated the proprioceptive drift during the RHI paradigm (Teaford et al., 2021). These findings seem to highlight that the embodiment of the RHI modulates arousal and sympathetic response of the autonomic nervous system. Such response may be suggestive of a bidirectional influence between autonomic nervous system and the sense of ownership: interoception modulates body ownership, via the afferent branch of the autonomic nervous system, and in turn, this modulation changes autonomic outflow, manifested through changes of sudomotor and vasomotor activity. Future studies are needed to validate this proposition, and to confirm that these modulations are not only an effect of experimental novelty. Indeed, participants’ interoceptive capability indexes, such as the scores in heartbeat counting tasks, seem to be not correlated to the strength of RHI (Crucianelli et al., 2018, Horváth et al., 2020). In addition, several indices, such as heart rate mean value and variability, as well as skin conductance peak frequency and amplitude are not affected by the RHI (Critchley et al., 2021). These main findings are summarized in Fig. 2 (Autonomic system measures).

## 7 Rubber hand illusion, embodiment and neuroprosthetics

### 7.1 RHI paradigm for studying embodiment in amputees and people with sensorimotor deficits

A key goal in rehabilitation engineering is to restore motor and sensory function of a lost limb with an artificial substitute that not only acts and feels, but is also felt as the biological one. To this end, RHI was one of the first paradigms employed to assess the embodiment of a prosthesis when afferent feedback from such prosthesis was provided to the user. In healthy individuals, it is critical to stimulate exactly the same locations on the rubber hand and the real hand for an illusion to be produced. In amputees, since there is not a hand to stimulate, some doubts can be cast on the possibility to elicit an illusion. However, when the hand representation in S1 is deafferented by the amputation, it is invaded by neighbouring areas such as the stump or the face (Lotze et al., 1999, Merzenich et al., 1984, Pons et al., 1991). This reorganization can justify phantom sensations referred to the missing digits elicited by touching the stump (Di Pino et al., 2021), which may also be exploited to elicit the RHI. Thus, the area of referred touch can be exploited for brush-stroking, but are amputees sensible to the illusion? Ehrsson and colleagues (Ehrsson et al., 2008b) demonstrated that, although to a lesser degree, the RHI can be elicited also in amputees. Moreover, similar cortical areas involved in multisensory processing, such as premotor and intraparietal cortices, are activated in both healthy individuals and amputees during the RHI (Schmalzl et al., 2014).

Several variations of the experimental setup exist where different tactile stimulations have been provided to the amputees (Fig. 1, Table 2). In addition to the classic brush stroking at the stump, vibrotactile stimulators have been used to convey tactile information on a group of transradial amputees, while participants saw the brushstrokes delivered on the digits of a fake hand (D’Alonzo et al., 2015). The demonstration that the RHI can also be elicited when different types of stimulation are applied to the subject’ limb and the artificial hand opened the field to a very interesting possibility; small devices such as electrotactile or vibrotactile stimulators, more easily embeddable in the prosthesis socket than a tapper, leveraging on sensory substitution could be used to convey sensory feedback related to the prosthesis, as well as to sustain its embodiment (D’Alonzo et al., 2015, Marasco et al., 2011, Pinardi et al., 2023). Indeed, in the future, a complete restoration of the upper limb would only be possible when the individual will sense the touch and the movement of their prosthesis, and feel it as a part of their body. Considering that self-attribution of a fake hand is mediated by multisensory perceptual correlations (e.g., a match between the afferent somatic signals and visual feedback from the hand), studies report that a lack of somatosensory-like feedback is one of the limiting factors affecting ownership of prosthesis (Beckerle et al., 2018), with the related feeling of interacting with a foreign body (Murray, 2004). This, in turn, may result in lower usage in daily activities and higher abandonment rate (Bekrater-Bodmann, 2021, Ostlie et al., 2012).

**Table 2:** Findings obtained in studies that apply variations of the RHI paradigm in amputees.

Study	Employed paradigm derived by RHI	Involved participants	Findings
Ehrsson et al. (2008b)	Brushstrokes of phantom fingers of the stump synchronously to a rubber hand	15 transradial amputees	Embodiment of fake hand can be elicited also in amputees
Rosén et al., 2009a	Brushstroke of the digits of prosthesis translated as brushstroke on the phantom fingers of the stump	4 transradial amputees and a participant with congenital missing hand	Illusion of embodiment elicited using touch on both prosthesis and stump
Marasco et al. (2011)	Touch of the digits of prosthesis translated as pressure on the targeted reinnervation skin	2 transhumeral undergone to TMSR	Illusion of embodiment elicited using touch on prosthesis and on reinnervated skin
Schmalzl et al. (2014)	Brushstrokes of phantom fingers of the stump synchronously to a rubber hand performed in MRI	2 transradial amputees	Same brain regions underlie ownership sensations of an artificial hand in amputees and non-amputees
D’Alonzo et al. (2015)	Vibrotactile stimulation on phantom fingers of the stump stimulated synchronously to the brushstroke of a rubber hand	9 transradial amputees	Illusion elicited when different types of stimulation are applied to the subject’ limb and the artificial hand
Sato et al., 2018	Open and close a robotic hand with a velocity proportional to the EMG amplitude recorded at the stump (motor hand illusion)	3 transradial amputees	Prosthesis embodiment elicited by the match between vision and the efference
Page et al., 2018a	3 tested conditions: tactile feedback provided to the prosthesis delivered by intraneural stimulation to the user, control of the prosthesis using intramuscular electrodes, and the combination of both conditions	1 transradial amputee implanted with intramuscular electrodes (iEMG) and Utah Slanted Electrode Arrays (USEAs)	Similar level of elicited prosthesis embodiment and phantom pain reduction among conditions
Rognini et al. (2019)	In VR environment, intraneural stimulation delivered synchronously with visual illumination of the region of the prosthesis corresponding to the referred location of the perceived touch	2 transradial amputees implanted with transverse intrafascicular multichannel electrodes (TIMEs)	Prosthesis embodiment and reduction of the phantom limb telescoping induced by visuotactile stimulation
Zbinden and Ortiz-Catalan (2021)	Touch of the digits of prosthesis translated as peripheral nerve stimulation	4 transhumeral amputees with osseointegrated human-machine gateway	RHI-induced embodiment and the long-term embodiment of a prosthesis may be mediated by (at least partially), different mechanisms

RHI has also been employed to test not only embodiment of a fake rubber hand but also a functional robotic device. In transradial amputees, RHI was elicited by touching the digits of prosthesis and translating this stimulation as pressure on the skin where the touch of the phantom hand was referred (Rosén et al., 2009a). The same paradigm was replicated in two transhumeral amputees who underwent a targeted muscle and sensory reinnervation (TMSR). The surgical approach for TMSR consists of rerouting motor and sensory nerves originally devoted to the lost hand, wrist and elbow towards intact muscles and skin regions (Kuiken et al., 2004, Kuiken et al., 2007), allowing to control the prosthesis by electromyography from reinnervated muscles and feed back the touch sensation on the missing limb by stimulation of the reinnervated skin areas. The reinnervated skin instead of the stump was stimulated and a whole integrated prosthetic platform was employed (Marasco et al., 2011). In these studies, motor control of the prosthesis was not assessed. To study the impact of motor control on prosthesis embodiment, Sato and collegues (Sato et al., 2018) proposed a modified version of the RHI, where three amputees were asked to continuously open and close a robotic hand with a velocity proportional to the EMG amplitude. Two conditions have been tested, one seeing the prosthesis movement synchronous to the muscle activation, and the other asynchronous. Sense of ownership, as measured via RHI questionnaire, was extended to the EMG-controlled robotic hand, with significant difference between the two conditions, highlighting the importance of the match between vision and the efference in prosthesis embodiment.

Recent technological developments allow prosthetic prototypes to be controlled and provide tactile feedback by invasive interfaces applied at central and peripheral level of nervous system of the users (Oddo et al., 2016, Raspopovic et al., 2014, Zollo et al., 2019). In amputees, placing an invasive interface at the level of the stump seems to be the best choice in terms of invasiveness and control performance, however other pathological conditions may require different approaches. For instance, in tetraplegic patient, direct and selective stimulation of the somatosensory cortex through invasive cortical interfaces have shown to be able to induce ownership of an artificial hand (Collins et al., 2017), and when the decoding of the motor command was added to the loop agency over the movement of a virtual hand was elicited as well (Serino et al., 2022). The RHI paradigm has been also employed to assess the embodiment of a prosthesis endowed with tactile feedback conveyed by a multielectrode array implanted in the stump peripheral nerves of a transradial amputee. Three conditions have been tested: tactile feedback alone, active movement alone, and the combination of both. All of those conditions successfully produced the illusion, with no better results of the latter, in prosthesis embodiment as well as in phantom pain reduction (Page et al., 2018a). Moreover, a modified version of RHI paradigm was employed in two transradial amputees implanted with multichannel intraneural electrodes capable of providing tactile-like stimulations. In VR environment, intraneural stimulation was delivered synchronously with visual illumination of the region of the prosthesis corresponding to the referred location of the perceived touch. The congruent visuotactile stimulation induced prosthesis embodiment, as well as the reduction of the phantom limb telescoping, which is sign of abnormal phantom limb perceptions (Rognini et al., 2019).

In several studies, even when the RHI paradigm itself was not applied, measures derived from it, e.g., questionnaire and proprioceptive drift, have been employed to assess embodiment or phantom limb representation (Graczyk et al., 2018, Rossini et al., 2010, Valle et al., 2018).

The RHI is the most widely used tool to study embodiment. However, recently, some doubts have been casted on the value of its translation to prosthesis embodiment; the experimental setup is structured and artificial, the fake hand is typically not worn and in most of the cases it cannot move, and more than anything else, the illusion is only temporary (D’Alonzo et al., 2020, Niedernhuber et al., 2018). Additionally, in recent study, Zbinden and Ortiz-Catalan (2021) reported that long-term users of prostheses capable to provide peripheral nerve stimulation were able to feel their prostheses as part of their body, but none of the them reported ownership over their prosthesis using a modified version of the RHI paradigm where synchronous tapping and peripheral nerve stimulation were employed. Since the participants did not report ownership also during the original RHI experiment in their contralateral hand, a potential explanation of the study's finding is that the participants are people not responding to the RHI (Zbinden and Ortiz-Catalan, 2021). This study demonstrates that, for such group of subjects, RHI paradigm cannot be employed to assess embodiment of prosthesis and suggests also that RHI-induced embodiment and the long-term embodiment of a prosthesis may be mediated by (at least partially), different mechanisms. However, it is worth to report also that only self- evaluation questionnaire was employed to probe the illusion; this is a limit of the study.

### 7.2 Cortical plasticity in amputees

From the evidence presented in the present article we can presume the known neural basis of RHI as the attenuation of primary sensory-motor processing to reduce the weight of somatosensory afference and efference copy (i.e., a copy of the motor command used to predict future sensory feedback) and an enhanced activity of the frontoparietal network to achieve the most meaningful multisensory integration notwithstanding the sensory mismatch.

To establish whether and how effectively the RHI paradigm can be used to monitor the embodiment of closed loop and highly-interactive prostheses in amputees, the knowledge of how primary sensory-motor activity and frontoparietal integration is affected by the amputation itself, and by the prosthesis use, is of help. Amputation results in changes in intra- and inter-network connectivity. An ICA-based fMRI connectivity analysis found that compared to healthy control, amputees showed decreased functional connectivity between sensorimotor network and fronto-parietal network, as well as between sensorimotor and dorsal attentional network (Bao et al., 2022). The previously mentioned decrease of primary sensory-motor representation (Pons et al., 1991, Merzenich et al., 1984), which is aimed to maximize the function of the residual limb, has been traditionally associated to maladaptive changes and correlated to phantom limb pain (Di Pino et al., 2009, Flor et al., 1995, Makin and Flor, 2020) (Flor et al., 1995, Di Pino et al., 2009). More recently, this view has been challenged, and the origin of phantom limb pain has been ascribed to several different causes acting at multiple levels of the central and peripheral nervous system (Di Pino et al., 2021).

Together with the changes triggered directly by the amputation, prosthesis usage counteracts the non-use of the deprivation-dependent hand sensorimotor loop affecting amputees brain activity as well. Indeed, the use of prostheses reduces the amount of amputation-related cortical reorganization (Lotze et al., 1999). When an amputee was trained with a sensorized prosthesis with neural interface, the aberrant intra-M1 plasticity, assessed with continuous theta burst stimulation (cTBS), tends to normalise, while the plasticity induced by the afference (inhibitory paired associative stimulation PAS) is disinhibited (Zollo et al., 2019). This study also showed that the selective stimulation of few nerve fibres is able to convey near-physiological feedback which can modulate M1 activity and drive the performance improvement (Ranieri et al., 2022). In another study, primary sensory-motor and integrative brain regions were assessed in patients who underwent a targeted muscle and sensory reinnervation (TMSR) but did not use sensorised prostheses. M1 and S1 activity and connectivity were almost normal, but their interplay with the frontal and parietal areas was highly impaired and was associated with reduced ability in prosthesis-related multisensory integration (Serino et al., 2017). This raises the intriguing question of whether the limitations of prostheses lacking sensory feedback, even of the most evolved, may still preclude a successful impact on the abnormal frontoparietal activity of amputees, attenuating the cortical maladactivity.

### 7.3 Alternatives to RHI paradigm to study embodiment of prosthesis

All protocols employed to assess prosthesis embodiment in amputees are based on how the prosthesis impacts on multisensory integration, because multisensory integration is known to be dependent on the relevance of the stimuli location for the body. One of this is temporal order judgment (TOJ; Yamamoto and Kitazawa, 2001). This is a two-alternative-forced choice experiment where participants have to state which of two stimuli delivered on the hand has been delivered first. When TOJ is tested with the hands crossed in healthy participants, the performance typically deteriorates because the spatial coordinates of the tactile somatotopic input come in contrast with the visual external coordinates. The expansion of the peripersonal space, the room around the body where stimuli matter more, is also a proxy of embodiment and can be tested by investigating the border of the area where the summation of two stimuli of different modalities produces a stronger reduction of the reaction time (Canzoneri et al., 2012). The space-body connections tested by the two protocols are partly different: a sensory-oriented embodiment is better assessed by the expansion of peripersonal space to include the prosthesis, while the crossing hand effect in the TOJ task is a more motor-oriented embodiment. Di Pino and collegues (Di Pino et al., 2020) recently showed in an amputee receiving sensory feedback through intraneural electrode that the anthropomorphism of the prosthesis matters for the former, but the latter is only achievable through a continuous training.

Another paradigm employed to study embodiment is sensory attenuation, a reduction of perceived intensity of a stimulus when the stimulus is self-generated. This can be assessed by a force-matching task where participants are requested to reproduce the force exerted on their finger by pressing a force sensor. In case of self-generated force stimulation, the reproduced force was lower compared to stimulation by others (Shergill et al., 2003). In able-bodied participants, the behavioural force-matching task was combined with the RHI paradigm to assess the attenuation effect. The more was the embodiment, the stronger the attenuation produced by a movement of the rubber hand and the weaker the one produced by a movement of the real, but disembodied, hand (Kilteni and Ehrsson, 2017). These findings demonstrated that sensory attenuation can be used as a proxy of embodiment because it is stronger when the effector used to produce the touch is considered a part of one’s own body. In amputees, touch exerted through the prosthesis produced a stronger attenuation compared to externally-generated touch, which correlated with the level of prosthesis embodiment (Fritsch et al., 2021). Implicit behaviour, such as co-speech gesticulation, have been also suggested as correlated to prosthesis embodiment and to its functional use proficiency (Maimon-Mor et al., 2020), suggesting an acquired transparency of the prosthesis for the phenomenal self (Makin et al., 2017).

## 8 Discussion- How does knowledge from the RHI translate to prostheses embodiment?

The aim of this article is to discuss neural and neurophysiological correlates of the rubber hand illusion, as a tool to study embodiment in healthy subjects and amputees. Since its seminal description (Botvinick, M., and Cohen, J. D, 1998), several variants of the RHI paradigm have been developed, to the extent to which Riemer and colleagues(Riemer et al., 2019) coined the term “rubber hand universe” to characterise the almost infinitive variations. Despite experimental heterogeneity, a consistent result is that the sense of ownership may be partly discernible from the simple response to the visuo-tactile conflict instantiated by the paradigm. Accordingly, converging evidence indicates that several cortical areas differentially contribute to particular aspects (e.g., ownership, self-localization) and behavioural measures (e.g., proprioceptive drift) of illusory embodiment. Premotor and parietal areas, which are consistently shown to be involved in the RHI, may have different roles in the embodiment of non-body objects (Guterstam et al., 2019, Kammers et al., 2009, Peviani et al., 2018), Specifically, parietal areas resolve visuo-tactile conflict arising from visual and tactile information, while premotor areas integrate the resulting feedback into the existing internal bodily representation (Limanowski, 2021). Studies suggest a somewhat equivalence in brain activity between healthy and amputees experiencing the RHI (Schmalzl et al., 2014). Furthermore, modulation in connectivity strength between visual and somatosensory areas with the parietal cortex follows the dynamic of recent computational accounts of predictive coding (Limanowski, 2021), and is coherent with a precision-based attenuation of somatosensory processing, resolving the visuo-proprioceptive conflict instantiated by synchronous visuo-tactile stroking (Lee and Chae, 2016, Limanowski and Blankenburg, 2015, Zeller et al., 2016). The visuo-tactile conflict is resolved by attributing visual stream a higher weight, compared to the proprioceptive one, which in turn is attenuated (Limanowski, 2021). This process is thought to rely on parietal processing (Limanowski and Blankenburg, 2015, Zeller et al., 2015, Zeller et al., 2016), which have been shown to be modulated by tactile input (Guterstam et al., 2019). Somatosensory attenuation may also explain why the MEP amplitude has found to be significantly smaller during synchronous compared to asynchronous brush stroking (della Gatta et al., 2016, Isayama et al., 2019). Attenuation of somatosensory information may induce a *disembodiment* of the real hand (Fossataro et al., 2018, Longo et al., 2008), in favour of embodiment of the rubber one, in order to resolve the visuo-proprioceptive conflict (Limanowski, 2021). In practice, this may result in a re-mapping of the hand representation– from the real to the rubber one. In support of this, even a slight neglect-like asymmetry of spatial attention might facilitate the process of embodiment of an artificial hand (Zeller and Hullin, 2018). Disembodiment of the real hand during the RHI, though rather low (Longo et al., 2008, Reader and Ehrsson, 2019), is also supported by the fact that corticospinal excitability significantly decreases with increasing stimulation time (della Gatta et al., 2016). These cortical mechanisms could extend to embodiment of prostheses by amputees. However, it needs to be kept in mind that post-amputation plasticity induces a re-organisation of neural substrates originally devoted to the now-missing body part. Nevertheless, studies show that amputees can embody a dummy hand, but its functional meaning with regard to prothesis use is yet to be fully explored. Until now, RHI studies suggest that, for an integration of an artificial hand into the amputee's own body schema, a physiologically feasible sensory pattern needs to be provided (Oddo et al., 2016, Ranieri et al., 2022, Raspopovic et al., 2014).

As already mentioned in the introduction, embodiment of an artificial hand includes both sense of agency (i.e., “it was me who moved that hand”) and ownership (i.e., “this hand is mine”). These two components depend on different perceptual and cognitive processes involving different cortical areas, whereas hand ownership is typically associated with activity in premotor, posterior parietal and cerebellar regions, while agency over the hand’s movements is related to activity in the dorsal premotor cortex and superior temporal cortex (Abdulkarim et al., 2023). Previous studies seem to show that agency can be perceived only when eﬀerent component is present, while ownership can arise also in its absence, but either visuomotor congruency (in case of agency) or inter-sensory congruency (in case of ownership) are needed to generate embodiment (Pinardi et al., 2020). For such reason, ownership and agency of the prosthesis can be elicited by just controlling it by using invasive or non-invasive interfaces (Sato et al., 2018, Serino et al., 2022), but it is not possible to induce agency if the moved prosthesis is not controlled by the user. For instance, studies using passive movement paradigms show that, without active movements, the sense of agency is absent (Kalckert and Ehrsson, 2012) or weakened (Desantis et al., 2011). On the other hand, the sense of agency can exist without body ownership, in such a way that interviewed amputees can report the feeling of agency regarding their prostheses but not that of body ownership (Wijk and Carlsson, 2015).

Sensorimotor or intersensory congruency (in case of agency or ownership, respectively) means not only synchronicity, but also small delays (i.e., < 500 ms) and resemblance to a preconceived model of the missing limb. Studies on healthy participants suggest that embodiment is relatively stable for inter-stimulation delays up to 300 ms (Shimada et al., 2009), above which embodiment is not found. However, more recent studies found a significant decrease in illusory hand ownership at delays or 150–200 ms (Chancel and Ehrsson, 2020; Chancel et al., 2022). To the best of our knowledge, no studies replicated this study in amputees. Similar sensitivities for delays are also found on the motor side. In studies employing the virtual hand illusion, controlled by participants’ movement, ownership of the virtual hand was reported if the delay between participants’ real movement and the visual feedback of the virtual hand had a delay between 90 and 190 ms. On the other hand, participants reported a strong sense of agency over the virtual hand for delays up to 490 ms, with stronger reports up to 190 ms. Using a similar setup, but with a different movement, (Shibuya et al., 2018) found that ownership of a virtual hand is retained up to 330 ms, decreasing thereafter, while agency was reported up to 450 ms. These results have direct implications for the development of prostheses. They highlight those technologies need to be developed such as to minimise the sensorimotor transmission delay. Interestingly, even in conditions of visuo-tactile incongruence, it is possible to induce embodiment of a rubber hand, as long as the stimulation is synchronous (Cipriani et al., 2011, D’Alonzo et al., 2015, Schütz-Bosbach et al., 2009). However, the level of elicited embodiment is lower with respect to congruent conditions (D’Alonzo et al., 2019).

The degree of anthropomorphism of the prosthesis is a debated issue. People can embody non-body objects of varying dimension (Bruno and Bertamini, 2010) and materials (Kalckert and Ehrsson, 2012; Rosén et al., 2009b; Pinardi et al., 2020). A now-classic work from Armel and Ramachandran (2003) suggested that it is possible to induce a sense of embodiment even in the absence of a dummy hand. If participants felt brush stroking and saw the stroking on a table, the embodiment was directed towards the table. However, the obtained results are not conclusive. Indeed, although they found a significant difference between level of induced illusion between synchronous and asynchronous condition, the induced level of ownership in synchronous stroking a table is similar to the no illusion condition with a rubber hand (i.e., asynchronous stimulation). Additionally, other studies did not confirm such finding (Tsakiris et al., 2010, Ehrsson et al., 2008a). In particular, one of them, where the appearance of the visible stimulated object was modified from a wooden block to a realistic rubber hand, found a sense of ownership only for the realistic prosthetic hand, suggesting that the viewed object must fit with a reference model of the body that contains important structural information about body parts (Tsakiris et al., 2010). Additionally, several studies seem to highlight the possibility to induce embodiment for both cosmetic and robotic prostheses in amputees (D’Alonzo et al., 2015, Ehrsson et al., 2008b, Page et al., 2018b, Rosén et al., 2009b). These findings seem to indicate that, to elicit a body ownership towards an object, it has to have the overall shape and fit with a human body part, which stands in contrast to research in virtual reality and augmented reality, where body ownership has shown to be much more plastic, enabling embodiment of avatars with very different shapes and looks (e.g., Roel Lesur, Aicher et al., 2020).

Visual information about the prosthesis has direct application for amputees. Indeed, prostheses are categorised differently compared to tools, but also biological limbs, which is reflected in activity in the occipitotemporal cortex (OTC) areas, as well as greater functional coupling between OTC and sensorimotor hand areas (van den Heiligenberg et al., 2017). OTC is known to contain distinct visual representations of different object categories, which has direct application to prostheses. A study by (Maimon-Mor and Makin, 2020) suggests that compared to controls, amputees categorise both cosmetic and active prostheses similarly, which, in turn, are categorised differently from tools (a wooden-spoon in their study) and real hands. This suggests that embodiment of prostheses by amputees requires some degree of resemblance to the biological limb, but this is flexible, and some deviations from a real resemblance may be possible.

After more than 30 years of behavioural and neuroscientific research on bodily illusions, however, one may ask *what’s next?* Historically, the RHI has been used as a tool to understand the sense of bodily ownership (Ehrsson, 2020, Tsakiris et al., 2010), however, it has been suggested to be a valuable tool in clinical contexts, too (Ramakonar et al., 2011). To be translated to clinics, studies should further explore individual differences in embodiment, especially how it changes throughout the life span (Table 3). This is of particular interest for embodiment of prostheses, as amputation may happen at any age, and preparation to these prostheses may differ. On the other hand, further studies are needed to shed light on the applicability of the RHI in amputees. Especially with the rise of other tools to study embodiment in amputees, studies should focus on building bridges between methods in order to create a multi-dimensional toolbox to study embodiment.

**Table 3:** Future direction for the rubber hand illusion.

Issue	Category	
Individual differences in illusory response		Phenomenal
Changes in illusory response throughput lifespan		Phenomenal
Improvements in behavioural tools to measure phenomenal changes in embodiment		Methodological
Phenomenal and neurophysiological after-effects of repeated application of the RHI		Methodological
Influence of different wording type on the RHI questionnaire		Methodological
Adapted experimental setups to target specific aspects of embodiment (e.g., disembodiment)		Methodological
